# Facial ambiguity and perception: How face-likeness affects breaking time in continuous flash suppression

**DOI:** 10.1167/jov.24.9.18

**Published:** 2024-09-27

**Authors:** Michael Makoto Martinsen, Kairi Yoshino, Yuya Kinzuka, Fumiaki Sato, Hideki Tamura, Tetsuto Minami, Shigeki Nakauchi

**Affiliations:** 1 Department of Computer Science and Engineering, Toyohashi University of Technology, Toyohashi, Aichi, Japan

**Keywords:** face-likeness, binary face, breaking time, implicit processing, visual stimuli perception

## Abstract

Previous studies have elucidated that humans can implicitly process faces faster than they process objects. However, the mechanism through which the brain unconsciously processes ambiguous facial images remains unclear. In our experiment, upright and inverted black-and-white binary face stimuli were presented in a two-alternative forced-choice location discrimination task combined with continuous flash suppression, a technique that suppresses visual stimuli perception using rapidly changing masks. The breaking time (BT) or the time required for a stimulus to be perceptually recognized was recorded for each face stimulus. The results showed that the BT for inverted grayscale images was significantly longer than that for upright grayscale faces, whereas the BT for upright and inverted binary faces did not reach statistical significance. A significant correlation between face likeness and BT was established after evaluating face likeness for each binary face stimulus, with high-face-like binary faces exhibiting shorter BT and low-face-like stimuli resulting in a more prolonged BT. Our results suggest that even an ambiguous object rated highly in face likeness can reduce the BT under implicit processing, indicating the possibility that facial parts such as the eyes and nose are subconsciously detected in ambiguous facial stimuli, enabling facial perception.

## Introduction

Research on face recognition has uncovered fascinating insights into the brain's unique facial processing mechanisms. The brain possesses a specialized system for detecting faces, supported by face-selective areas and cells (Tsao & Livingstone, 2008) that integrate facial data with physical characteristics. This system plays a crucial role in the early development of the social brain (Freiwald, 2020; Shoham, Kliger, & Yovel, 2021). Critical aspects of face individuation, such as encoding new faces (Haxby et al., 1996), holistic processing (Richler & Gauthier, 2014), and face-space effects (Valentine, Lewis, & Hills, 2015), are present as early as three to five years of age and sometimes even in infancy (McKone, Crookes, Jeffery, & Dilks, 2012).

Given their crucial role in human communication, faces and face-like stimuli are processed more thoroughly by the visual system even when not consciously perceived. One potential mechanism that involves the processing of partial visual facial information under unconscious conditions is the fusiform face area (FFA), which is considered to play a key role in holistic face processing (Richler & Gauthier, 2014; Zhang, Li, Song, & Liu, 2012; Zhou et al., 2018). The FFA may integrate minimal facial cues, such as the eyes and mouth, to form a coherent representation of a face even when visual information is limited (Jiang & He, 2006; Schwiedrzik, Melloni, & Schurger, 2018). Additionally, top-down processing mechanisms, wherein the brain uses prior knowledge and expectations to fill in missing details, may enhance the detection of faces under these conditions (Latinus & Taylor, 2005). Studies using EEG have shown that the N170 component, associated with face recognition, is activated by Mooney faces perceived as faces, suggesting early perceptual processing of facial information (George, Jemel, Fiori, Chaby, & Renault, 2005). Furthermore, the involvement of subcortical pathways, such as the superior colliculus and pulvinar, may facilitate rapid, unconscious detection of faces by directing attention to face-like stimuli (Tamietto & Gelder, 2010).

Building on the foundation of the brain's remarkable face recognition capabilities, we question how the brain perceives faces when presented with limited or incomplete visual information. The human ability to perceive faces does not rely solely on complete visual information. Using Mooney face stimuli (Schwiedrzik et al., 2018), which are binary face-like stimuli that evoke a shadowed half-face impression associated with limited facial information (Ke, Yu, & Whitney, 2017; Latinus & Taylor, 2005; Schwiedrzik et al., 2018), previous research has suggested that when the shadowed areas of an image are completed with cues from recognizable facial features, the brain perceives these images as faces. Moreover, EEG studies have shown that when a Mooney face is subjectively considered more of a “face” rather than a random object, the N170 component, strongly associated with face recognition, is detected. Contrarily, the effect disappears when the stimuli are flipped upside down, suggesting an inversion effect, a phenomenon highly linked to face perception (George et al., 2005).

Given their crucial role in human communication, the question arises: are faces and face-like stimuli processed more thoroughly by the visual system even when not consciously perceived, or do they receive superficial processing similar to other images? Researchers have adopted a technique called continuous flash suppression (CFS) to determine how visual stimuli are processed subconsciously. In this technique, a single visual stimulus is presented to one eye, while the other eye is presented with dynamic high-contrast flashing mask stimuli for a short period, causing interocular suppression and rendering the target stimulus perceptually invisible (Gayet, der Stigchel, & Paffen, 2014a; Gayet, der Stigchel, & Paffen, 2014b, Gayet et al., 2017; Tsuchiya & Koch, 2005; Yang & Blake, 2012). After a few seconds (or even two to 20 second), the interocular suppression is broken, enabling the participant to perceive the target stimuli again. The duration for the target stimulus to be seen again is often referred to as the breaking time (BT) and is used as a marker for processing efficiency; a shorter BT indicates that the target is more efficient in breaking through suppression than one with a longer BT.

Using this technique, studies have demonstrated that specific categories such as upright faces are recognized more efficiently than inverted faces are (Jiang, Costello, & He, 2007; Stein, Reeder, & Peelen, 2016) without conscious awareness, suggesting that brain regions such as the occipital face area and FFA (Yovel & Kanwisher, 2005) are implicitly activated. Other categories, such as human bodies and silhouettes, have also been reported to be recognized more efficiently than inanimate objects are (Stein, Sterzer, & Peelen, 2012). One study found that fearful facial expressions are more effective and demonstrate shorter BT than neutral or happy expressions, even when faces are inverted or only the eye regions of different facial expressions are displayed (Yang, Zald, & Blake, 2007). These empirical findings support the notion that visual processing may confer certain advantages to facial stimuli. However, how images with low information content, in which the contours, eyes, and mouth are missing, are processed in an unconscious state is yet to be clarified.

Based on these findings, we hypothesize that the lack of information in binary face stimuli will hinder holistic processing and potentially compromise detection efficiency, leading to different results from those for gray face stimuli. We anticipate that stimuli with a higher face likeness will elicit faster detection times, reflecting the impact of resemblance on subconscious facial perception. Using the CFS, participants were presented with a series of suppressed facial stimuli to test this hypothesis. In the experiment, upright and inverted binary faces were used as the main visual targets, and upright and inverted gray faces were used as controls. The BT was recorded to examine the speed and accuracy of face recognition under different stimulus conditions.

## Methods

### Participants

Twenty-four students (22 male, 2 female) between the ages of 20 and 24 (M = 21.7, SD = 1.2) were recruited from Toyohashi University of Technology. The sample size was determined based on previous Mooney face (Canas-Bajo & Whitney, 2022; Takahashi & Watanabe, 2015) and CFS (Madipakkam, Rothkirch, Wilbertz, & Sterzer, 2016; Stein, Hebart, & Sterzer, 2011) studies, which recruited 24 participants. All students reported having normal or corrected-to-normal vision and participated in the experiment in exchange for payment (approximately 1000 JPY per hour). The experiments were approved by the Ethics Committee for Human Research at Toyohashi University of Technology and were strictly conducted according to the approved guidelines of the Committee and the Declaration of Helsinki. Written informed consent was obtained from the participants after the experimental procedures were explained.

### Apparatus

Face stimuli were presented on a liquid crystal display monitor (VIEWPixx/3D; VPixx Technologies Inc., QC, Canada) with a resolution of 1920 × 1080 pixels and a refresh rate of 120 Hz, which was explicitly optimized for stereoscopic visual target stimuli. Participants wore active shutter 3D glasses (3D Vision Wireless; Corp., Santa Clara, CA, USA) that displayed faces and masking stimuli to different eyes. The viewing distance was set at 80 cm, and a head-and-chin rest was used to stabilize participants’ fixation. For all tasks, participants used a millisecond latency keypad (MilliKey MH-4; LabHackers Research Equipment, Halifax, NS, Canada). The entire CFS experiment was controlled by software programmed in MATLAB R2020b (MathWorks, Natick, MA, USA) using Psychtoolbox-3 (Kleiner, Brainard, & Pelli, 2007; Pelli, 1997a, Pelli, 1997b) on a Microsoft Windows PC to present the stimuli and record the participants’ responses.

### Stimuli

In this study, grayscale and binary facial stimuli were created using MATLAB. Face images were randomly selected from the *Face Detection Dataset and Benchmark* database (Jain & Learned-Miller, 2010), initially cropped to 2.6° × 3.5° and converted to grayscale using the *rgb2gray* function. After applying a Gaussian smoothing filter with the *imgaussfilt* function (sigma set to 5.5) to each grayscale image, they were converted into white and black binary images using the *imbinarize* function. Previous research has shown that low-level features, such as differences in the number of pixels, can influence the BT (Heyman & Moors, 2014; Martinsen, Kinzuka, Sato, Minami, & Nakauchi, 2023); to minimize the effects between each face stimulus, we selected 20 binary images with a black over white pixel ratio ranging between 0.9 and 1.1 from the converted image pool.

### Procedure and task

**Figure 1.**
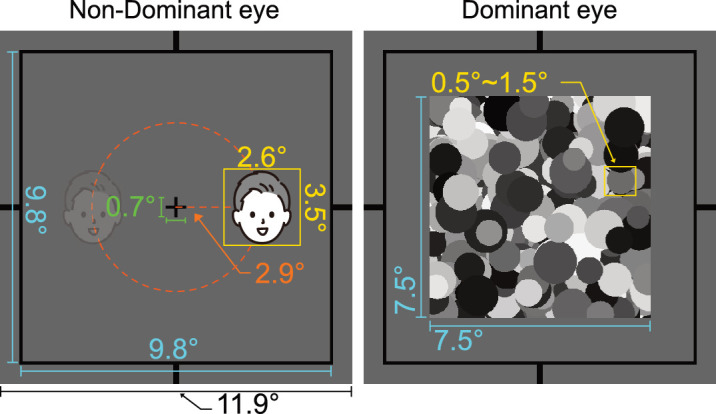
CFS experiment screen configuration. A gray rectangle (11.9° × 11.9°, outer line width: 0.09°) was used as background, then superimposed with a black frame (9.8° × 9.8°) to enable stable binocular fusion. A fixation (0.7° × 0.7°) was presented in the center of the screen for both eyes. Throughout the experiment, face stimuli were introduced to the nondominant eye. The face stimulus was positioned 2.9° away from the center and placed either on the left or right of the fixation. All face stimuli were cropped and resized to fit 2.6° × 3.5°. A dynamic flashing mask stimuli was presented to the dominant eye. Each masking stimulus consisted of randomly generated gray circles, sized between 0.5° and 1.5° with random luminance. The positions of each square were generated to fit in a 7.5° × 7.5° window centered at fixation.

During the experiment, a fixation cross (0.7° × 0.7°) was placed at the beginning of each trial and the participants were instructed to focus on it (Figure 1). Although a facial stimulus was presented to the non-dominant eye, masking stimuli were introduced to the dominant eye at a rate of 10 Hz to enable perceptual suppression. Prior CFS experiments have conventionally utilized this refresh rate for masks; therefore, we adopted it in this study (Martinsen et al., 2023; Pournaghdali & Schwartz, 2020; Tsuchiya, Koch, Gilroy, & Blake, 2006; Tsuchiya & Koch, 2005). Additionally, each masking stimulus consisted of randomly generated gray circles, sized between 0.5° and 1.5° with random luminance because face target stimuli work well with circular rather than rectangular masks, and the masking stimuli should also be black and white and not chromatic for grayscale and black/white targets (Pournaghdali & Schwartz, 2020). The positions of each square were generated to fit into a 7.5° × 7.5° window centered at the fixation.

**Figure 2.**
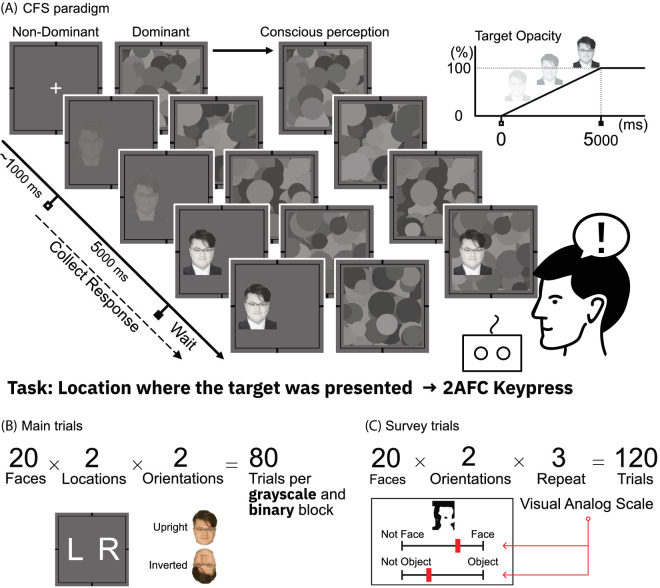
The CFS experiment paradigm. (**A**) A dynamic flashing pattern is presented to one eye every 10 Hz. Concurrently, a random face stimulus was introduced to the other eye. In this period, the face stimulus opacity linearly increased from 0% to 100% in five seconds, then remained constant until the participant responded or reached the maximum time limit set at 15 seconds. Participants were instructed to answer the location (left or right) of the face, which emerged into awareness through a two-alternative force-checked location discrimination task. The face sample used for this graph is that of MMM, the first author of this paper. (**B**) For both grayscale and binary conditions, twenty faces were selected as target stimuli. Each face stimulus was presented to the left and right, and additionally, upright and inverted. Therefore, participants conducted a total of 80 trials for each condition. (**C**) After both experiment blocks, participants evaluated each binary image's subjective face-likeness and object-likeness from 1 (left) to 5 (right) using a visual analog scale. In this scale, 5 indicated high face-likeness or object-likeness, whereas 1 indicated the opposite.

Figure 2 illustrates the procedure for each trial. The ∼1000 ms interval represents the initial presentation of masking stimuli without the target stimulus to enhance suppression effectiveness. Following this interval, the face stimulus opacity was increased linearly from 0% to 100% within five seconds and remained constant for another 10 seconds (Figure 2). From stimulus onset to 15 seconds, the participants were instructed to identify the face stimulus location (left or right) that emerged through awareness through a two-alternative force-checked task as fast as possible using the keypad. In the main task, the participants were not required to indicate the stimulus orientation. Throughout the experiment, the participants were instructed to maintain a stable fixation at the center of the monitor during each trial. The order of target stimulus presentation was randomized for each participant throughout all trials in both blocks based on location (left/right) and orientation (upright/inverted). To minimize familiarity affecting the BT against binary stimuli, all participants first conducted the binary stimuli block and were subsequently instructed to conduct the grayscale-stimuli-condition block in the CFS experiment. The total number of trials per block was 80 (20 Face Stimulus × 2 Locations × 2 Orientations); therefore the entire experiment comprised 160 trials. After completing both blocks, the participants evaluated face likeness and object likeness for each binary face stimulus, as illustrated in Figure 2(C). For the evaluation survey, each participant completed 120 trials (20 Binary Faces × 2 Orientations × 3 Repeat). Using a visual analog scale, the participants evaluated each binary image's subjective face and object likeness between one (left) to five (right). Here, five indicates high face or object likeness on this scale, whereas one indicates the opposite.

### Data analysis

The BT occurred when the participants pressed a button and correctly identified the location of the facial stimulus. The analysis only considered trials in which participants accurately detected the target when it was presented alongside a masking stimulus. The excluded trials consisted of instances in which participants indicated an incorrect target location, responded too quickly (within 100 ms) after target presentation, or responded after the masking stimulus disappeared (15 seconds or more). Trials that exceeded three standard deviations from the mean were excluded. Overall, 8% of trials were excluded. Five participants were rejected based on their poor performance (incorrect trials, 30%) in localizing the face stimuli. Participant's mean BT were calculated for each experiment condition, then we conducted a paired *t*-test for each block to analyze the effect of location and orientation in R (version 4.2.3). Correlation analysis between the face-likeness, object-likeness, and BT were performed using linear regression in R. In addition, to consider between-subjects variance, we analyzed BT using a linear mixed model with participants and face stimuli ID as random effects. For fixed effects, face-likeness (1 to 5), object-likeness (1 to 5), and orientation (−1 as inverted, 1 as upright) were used. The mixed model statistics and degrees of freedom were estimated using the R package “lmerTest,” and the step function was used to determine the best fit model.

## Results

### Binary facial stimuli do not produce the inversion effect

**Figure 3.**
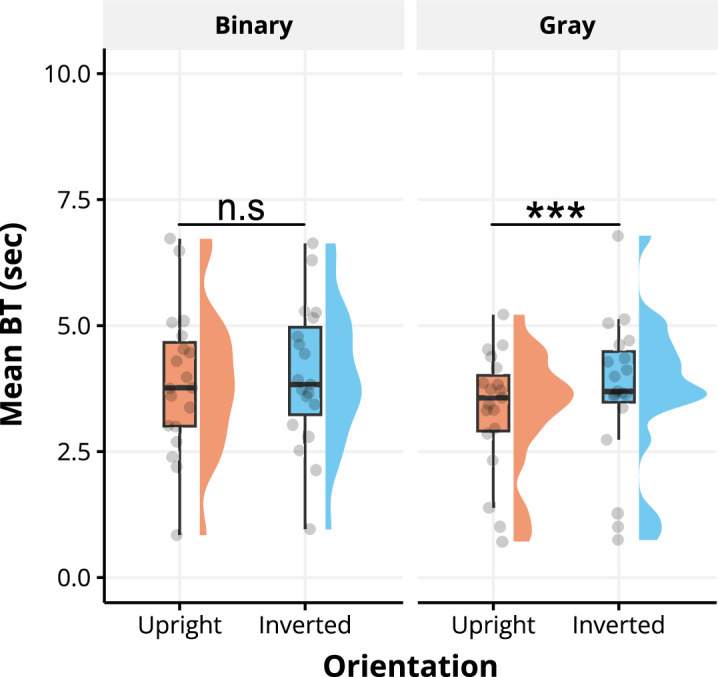
BT for each experiment block (binary and gray). The orange bar indicates upright face condition and the blue bar indicates inverted face condition. Each bar shows the mean BT and each error bar indicates standard error. Asterisks indicate a significant difference between upright and inverted gray face experiment block (*** *p* < 0.001).

Paired *t*-tests were conducted to compare the mean BT between upright and inverted faces within the binary condition and the binary block individually. Figure 3 shows mean BT for each orientation and block condition. Prior to conducting the *t*-tests, the assumption of normality and homogeneity of variance were assessed. For the grayscale block, the Shapiro-Wilk test confirmed the normality of the distribution for upright (*W* = 0.92, *p* = 0.129) and inverted (*W* = 0.92, *p* = 0.120) faces. Levene's test indicated that the assumption of homogeneity of variances was also met between upright and inverted condition (*F*(1, 36) = 0.20, *p* = 0.654), within the grayscale block. The same analysis was conducted for the binary block. Shapiro-Wilk test confirmed the normality of the distribution for upright (*W* = 0.98, *p* = 0.953) and inverted (*W* = 0.99, *p* = 0.991) faces. In addition, the assumption of homogeneity of variances was also met (*F*(1, 36) = 0.00, *p* = 0.997). Given the normality assessment, we proceeded with a paired *t*-test.

**Figure 4.**
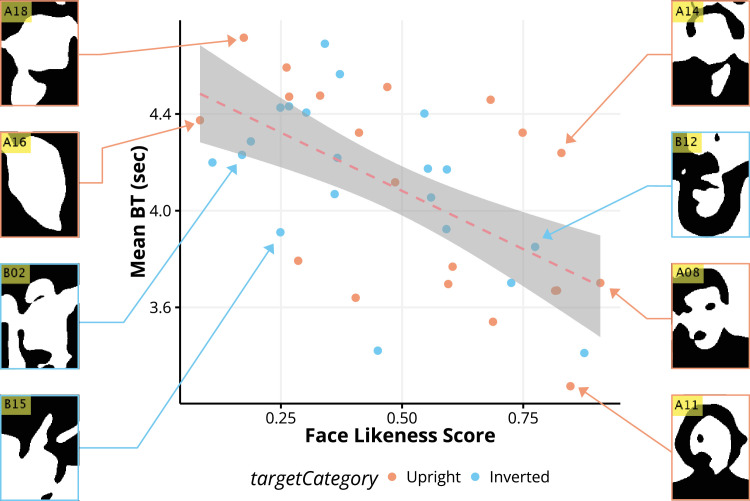
Correlation analysis between the face-likeness score (0 = not a face, 1 = face) provided through the survey, and average BT. Each dot represents a single binary face stimulus, where orange (blue) indicates that the stimulus was upright (inverted). There was a significant correlation between BT and the likeness (*r* = −0.59, *R*^2^ = 0.35, *p* = 5.80 × 10^−5^), indicating that when the likeness increased, BT reduced.

The paired *t*-test for the grayscale stimuli indicated a significant difference between the upright and inverted (*t*(18) = 4.22, *p* < 0.001, Cohen's *d* = 0.22) faces. However, BTs for upright and inverted binary faces were nonsignificant (*t*(18) = 1.61, *p* = 0.125, Cohen's *d* = 0.07), which can be explained by the lack of information to trigger face perception for the upright conditions. This significant difference between the upright and inverted conditions for grayscale face stimuli is consistent with the results of Jiang and Stein, who established that identifying upright faces was faster (Jiang et al., 2007; Stein et al., 2012, Stein et al., 2016). To explore the connection between the subjective face-likeness of binary faces and the BT required for face stimuli to break suppression, a correlation coefficient was computed between the BT and face-likeness judgment for upright binary and inverted binary faces separately (Figure 4).

### Face-likeness affects BT

**Figure 5.**
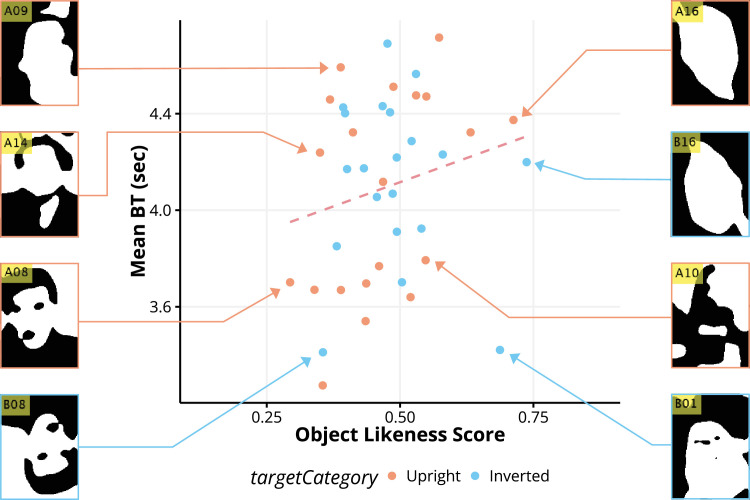
Correlation analysis between the object-likeness score (0 = not an object, 1 = object) provided through the survey and average BT. Each dot represents a single binary face stimulus, where orange (blue) indicates that the stimulus was upright (inverted). There was no significant correlation between BT and the likeness (*r* = 0.13, *R*^2^ = 0.02, *p* = 0.41).

The results revealed a significant correlation with face-likeness judgment in binary faces, (*r* = −0.59, *R*^2^ = 0.35, *p* = 5.80 × 10^−5^). When taking a closer look at each binary face stimulus, binary faces that resembled contours, eyes, mouth, and hair were judged to have high face likeness and relatively shorter BT, whereas unrecognizable shapes were judged as having low face likeness and longer BT. The same analysis was conducted for the object likeness, as illustrated in Figure 5.

**Figure 6.**
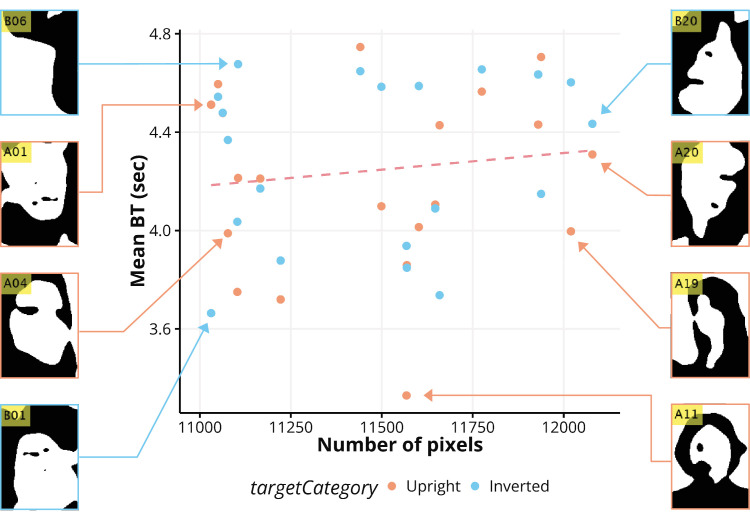
Correlation analysis between the number of black pixels in each binary face stimuli and averaged BT. There was no significant correlation between BT and the pixels (*r* = 0.13, *R*^2^ = −0.02, *p* = 0.41), suggesting that differences in pixels did not affect the detection speed of binary faces.

Interestingly, there was no significant correlation between the object-likeness of the binary faces and BT (*r* = 0.13, *R*^2^ = 0.02, *p* = 0.41) as shown Figure 5. Although a high correlation between face-likeness and BT exists, the number of pixels may have affected the BT. To investigate the influence of the pixels, the correlation was assessed via a scatter plot, as illustrated in Figure 6. There was no significant correlation between BT and pixels (*r* = 0.13, *R*^2^ = −0.02, *p* = 0.41), suggesting that differences in pixels did not affect the detection speed of binary faces. Considering previous research on CFS, our findings indicate that, even when suppressed and invisible, stimuli associated with higher face likeness undergo distinct processing compared to those associated with lower face likeness and unrecognizable forms. This processing advantage might have contributed to their ability to achieve dominance.

### Linear mixed-model analysis: Face and object likeness influence BT

The above results notwithstanding, there are several concerns about the data. A certain number of trials were excluded, resulting in the removal of several participants. As a result, there is a possibility that BTs for some stimuli were based on the responses of a subset of participants. To accommodate between-subject variance and ensure the robustness of our findings, a linear mixed-effect model was used for the following analysis.

Initially, multiple complex models were tested to explore the interactions between face-likeness (Face), object-likeness (Object), and category (Category), incorporating participants (subID) and face stimuli (targetID) as various random effects. The initial model was:

(1) $$\begin{array}{c} \begin{array}{c} BT\;∼Face\;\times Object\;\times Category\;\\ +\;\left.\left(1\;+\;Face\;\times Object\;\times Category\;|\;subID\right.\right)\\ +\;\left.\left(1\;+\;Face\;\times Object\;|\;targetID\right.\right).\; \end{array}\; \end{array}$$

This comprehensive model aimed to capture all possible interactions and random effects within subjects (subID) and targets (targetID). However, the model resulted in singularity errors, indicating overfitting and issues with model estimation due to the high complexity relative to the data structure. To address these issues and resolve the singularity errors, variables were manually removed step-by-step until a model without singularity errors was achieved. The simplified model was then:

(2) $$\begin{array}{c} BT\;∼Face\;\times Object\;\times Category\;\\ +\left.\left(1\;+\;Face\;|\;targetID\right.\right).\;\; \end{array}$$

For the next step, the best model was chosen based on a stepwise backward elimination process, optimizing for model fit and parsimony. Ultimately, the final selected model was:

(3) $$\begin{array}{c} BT\;∼Face\;+\;Object\;+\;\left.\left(1\;|\;targetID\right.\right).\;\; \end{array}$$

**Figure 7.**
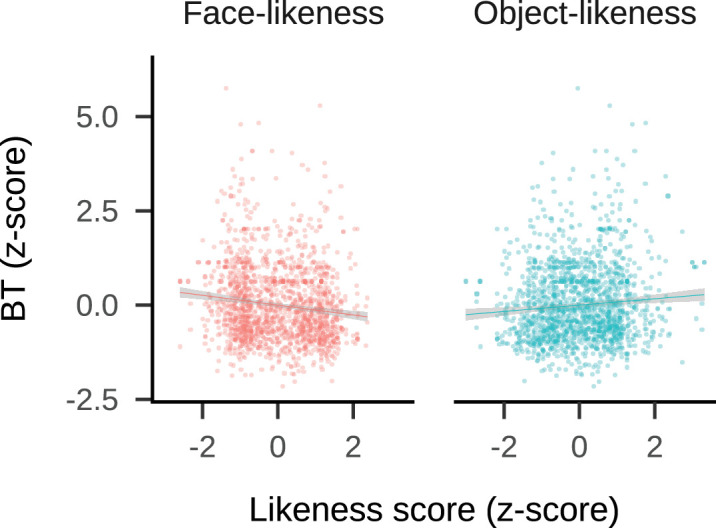
Relationship between Likeness Score and BT. Scatter plots showing the linear regression of likeness scores (*z*-scores) against BT (*z-*scores) for Face-likeness and Object-likeness. Regression equations: Face-likeness *y* = 0 – 0.13*x*; Object-likeness *y* = 0 + 0.84*x*. Shaded areas represent 95% confidence intervals.

In summary, the linear mixed model was fitted using restricted maximum likelihood (REML) to investigate the effects of face-likeness (Face) and object-likeness (Object) on BT, with random intercepts for targetID (40 groups, 1760 observations). The model's REML criterion at convergence was 4,950.18, indicating a good fit to the data after resolving singularity issues and simplifying the model to enhance stability and interpretability. This analysis revealed that as facial likability increases or object-likeness decreases, BT decreases. Detailed model results are presented in Table 1 and Figure 7.

**Table 1.** Summary of the Linear Mixed-Effects Model for BT Including Fixed and Random Effects. This table summarizes the results of the final linear mixed-effects model examining the influence of face-likeness (Face) and object-likeness (Object) on BT. The model includes fixed effects for Face and Object, as well as a random intercept and slope for targetID. The 95% confidence intervals (CI), *t*-values, degrees of freedom (*df*), and *p*-values are provided for each fixed effect. The model's REML criterion at convergence was 4,950.18.

Term	$\hat{\beta }$	95% CI	t	df	p
(Intercept)	0.00	[− 0.06, 0.06]	0.00	27.83	>0.999
Face	−0.09	[− 0.14, − 0.03]	−3.16	140.14	0.002
Object	0.05	[0.00, 0, 10]	2.05	1489.16	0.041

Figure 7 illustrates the relationship between likeness scores (*z*-scores) and BT (*z*-scores) for both face-likeness and object-likeness. The shaded areas around the regression lines represent 95% confidence intervals. The negative slope of the regression line for face-likeness indicates a significant negative correlation, suggesting that as the face-likeness score increases, BT decreases. This implies stimuli perceived as more face-like break suppression faster. Conversely, the positive slope of the regression line for object-likeness indicates a slight positive correlation, suggesting that as the object-likeness score increases, BT also increases, although the effect is weaker compared to that of face-likeness. These results may suggest the distinct influence of facial cues in accelerating perceptual awareness, highlighting the brain's specialized sensitivity to face-like features even under conditions of limited visual information.

## Discussion

The experiment revealed a significant difference in BTs between upright and inverted faces for grayscale images, consistent with previous studies (Gayet et al., 2014a; Jiang et al., 2007); however, the difference in BTs did not reach significance in the case of upright versus inverted binary faces. This suggests that the inversion effect typically observed in face perception was present for grayscale but not binary faces, indicating that binary facial information is insufficient to render facial perception unconscious. Further analysis revealed that binary faces with high face-likeness scores had significantly shorter BTs, suggesting that even minimal facial cues facilitate faster access to conscious awareness. However, there was no significant correlation between object-likeness and BT, indicating that object-like features do not have the same facilitative effect on perceptual awareness as face-like features. Additionally, our analysis showed no significant correlation between the number of pixels in the binary images and BT, suggesting that differences in pixel count did not affect detection speed. This finding rules out low-level visual features as a confounding factor in our results. To ensure the robustness of these findings, we employed a linear mixed-effects model (LMM) to account for between-subject variance. The LMM revealed that as face-likeness increases or object-likeness decreases, BT decreases. This highlights the distinct influence of facial cues in accelerating perceptual awareness, emphasizing the brain's specialized sensitivity to face-like features even under conditions of limited visual information.

Our findings align with the work of Caruana and Seymour (2022), who reported that objects inducing facial pareidolia elicit faster BTs. However, our study extends their findings by showing that the relationship between face-likeness and BT is not confounded by the number of pixels, thereby reinforcing the specific role of facial features in unconscious processing. The absence of an inversion effect for binary faces suggests that the lack of detailed visual information in these stimuli may hinder holistic processing. This aligns with the suggestion that our results could indicate “limited facial information in the binary faces” rather than “a limitation in unconscious facial perception.” To clarify this, future studies could analyze the subset of binary faces with the highest face-likeness scores to see if they exhibit an inversion effect.

These results suggest that the lack of information on binary face stimuli may have hindered holistic processing or partial face detection because of individual differences (Canas-Bajo & Whitney, 2020). Stimuli with higher face likeness were significantly correlated with faster BT when comparing their face likeness to the average BT per binary face stimulus, as illustrated in Figure 4. Previous studies have suggested that some unconscious facial information may reach high-order visual processing areas, as revealed by neuronal activity in the FFA (Jiang et al., 2009; Jiang & He, 2006; Sterzer, Jalkanen, & Rees, 2009). Visual information that could reach the FFA likely exerts a powerful influence in overcoming suppression and reaching conscious awareness compared to other stimuli that recorded slower BT.

Recognizing that using binary face stimuli is a relatively new approach in CFS face-processing research is imperative. Researchers have established that Mooney faces are processed holistically (Latinus & Taylor, 2005) and adapted to the N170 component, indicating a link between the N170 and holistic face processing (Eimer, Gosling, Nicholas, & Kiss, 2011) for ambiguous face stimuli. The N170 signal against Mooney faces was enhanced when primed with familiar faces. The fact that the priming effect was only observed with familiar faces and manifested at the N170 peak implies that top-down effects influence the early perceptual processing of faces (Jemel, B., Pisani, Calabria, Crommelinck, & Bruyer, 2003). This suggests that similar mechanisms may influence the recognition of binary faces. Therefore results obtained from earlier studies that used different stimuli should be interpreted cautiously. Understanding these mechanisms is crucial for interpreting findings in CFS face-processing research. This leads to the critical question: How early does this effect emerge?

Our brains possess an extraordinary capacity to extract crucial information from our surroundings, exemplified by our ability to perceive facial features in simple shapes, a phenomenon referred to as facial pareidolia or simulacra perception. The degree to which these stimuli resemble a face can significantly influence this phenomenon, and it is believed that these perceptions can subconsciously affect object and face perceptions. Supporting our findings, Caruana and Seymour (2022) reported that objects inducing facial pareidolia elicit faster perceptions. This finding is consistent with the hypothesis that the subcortical mechanisms responsible for detecting faces operate automatically and outside the realm of conscious awareness. This mechanism has evolved to prioritize rapid responses to faces, potentially at the cost of occasionally perceiving non-facial stimuli as faces, also known as “false positives” (Caruana & Seymour, 2022).

Another possibility is that the classification is done at an earlier stage rather than considering that FFA is processing ambiguous and rough information. Studies have found significant correlations between the inversion effect index and face-like scores in various components of the event-related potential, such as N170 and P1. This indicates that the neural processing of rough faces (detecting the mouth and nose shape) may be performed in P1, followed by N170, which is known to process facial details (Nihei, Minami, & Nakauchi, 2018). In addition, the amplitude of P1 has been reported to increase when presented with ambiguous visual stimuli (Dering, Martin, Moro, Pegna, & Thierry, 2011; Schupp et al., 2008). This implies that selective processing may occur in areas near the early visual cortex against rough amounts of information in a binary image, even before the FFA can perform advanced face identification. Of course, we must consider that the P1 component is sensitive to luminance contrast (O'Hare, Goodwin, & Sharman, 2023), suggesting that the pixel differences in our stimuli may have influenced BT rather than being an essential marker of early processing of visual stimuli. Nonetheless, our analysis revealed no correlation between the number of black pixels and BT, effectively eliminating the possibility that low-level features influenced the observed results, as suggested in previous research.

There is ongoing debate about the extent and nature of unconscious high-level processing, and studies using CFS often contribute to this debate with contentious or ambiguous findings. This is because results obtained using CFS might be influenced by factors such as attention, eye movements, or low-level visual processing, which complicate the interpretation of high-level perceptual processing. Nevertheless, a review by Sterzer, Stein, Ludwig, Rothkirch, and Hesselmann (2014) indicates that complex visual information, including object categories and emotional expressions, can still be processed in higher-level visual areas even when the stimuli are suppressed from awareness. For instance, the amygdala shows differential responses to emotional faces (e.g., fearful vs. neutral) regardless of their visibility (Sterzer et al., 2014). This indicates that certain aspects of face processing, particularly those related to emotional or social cues, can occur without conscious awareness. Shadows, specifically facial shadows, can be considered social cues as they enhance the perception of expressions and emotions, influencing the interpretation of someone's mood or intentions in social interactions. A recent study by Gelbard-Sagiv, Faivre, Mudrik, and Koch (2016) demonstrates that high-level processing, such as face identity recognition, is significantly influenced by the conscious perception of low-level features like color or location (Gelbard-Sagiv et al., 2016). Additionally, using Mooney faces, Peterson et al. (2023) discussed that our visual system is quite “tolerant” of detecting faces from a large variation of contrast patterns. Combining these findings, we suggest that the visibility of low-level aspects, such as contrast cues generated by the binary face shadows, may enable or enhance the visual processing of more complex, high-level information. This indicates that even when visual information is minimal, the brain can utilize these cues to facilitate higher-level perceptual processing.

Regardless of the specific mechanisms involved in transmitting information from the suppressed image to the face-selective regions, the primary outcome of this study indicates that visual stimuli resembling Mooney and binary faces may have the potential to activate specific cortical areas associated with facial recognition. In addition, we suggest there lies a delicate balance between sensitivity and specificity in facial recognition, leading to occasional misinterpretation of non-face stimuli as faces. A similar CFS paradigm, discussed by Jiang and He, revealed that even when participants were unaware of the content of the presented images, the FFA consistently exhibited more robust activation in response to invisible faces than to invisible scrambled faces (Jiang & He, 2006). These findings prove suppressed and imperceptible face-like stimuli evoke neural representations within face-specific cortical areas.

Our study contributes to the growing knowledge of face perception by investigating the processing of upright and inverted faces under CFS. Furthermore, our exploration of stimulus characteristics provides valuable information on the impact of face likeness and the potential role of holistic processing in subconscious face perception.

## Limitations and future works

Our study aims to deepen the understanding of the unconscious mechanisms involved in facial perception, especially under CFS, indicating that the brain can extract and process facial information from minimal cues, a trait likely evolved for the swift and effective recognition of faces. However, there are several limitations which must be addressed.

First, our study did not address the potential influence of other factors, such as emotion or attractiveness, which previous studies have shown to affect face perception (Li et al., 2019; Schindler & Bublatzky, 2020; Yang et al., 2007). If accomplished, isolating specific impacts of face-likeness on BT can be addressed specifically.

Second, the potential issue of stimulus-mask confusion should be addressed. For example, the extended BT for Mooney faces, or even the absence of any significant effect, might indicate a ceiling effect. This may be caused by a similar trend in spatial frequency distribution, suggesting that Mooney images and masked images share comparable spatial frequency characteristics. One idea is considering the usage of colorful Mondrian masks to avoid a partial piecemeal perception of the target stimuli, ensuring that the observed effects are genuinely due to the facial features of the stimuli and not an artifact of the masking technique.

Third, the variability in how participants interpreted inverted Mooney faces may pose another limitation. A correlation analysis between face-likeness ratings for upright and inverted Mooney faces revealed significant positive correlations for some images, indicating that participants perceived face-like features in both orientations. However, other images had weaker or non-significant correlations, suggesting that participants may have perceived less recognizable features or random blobs in the inverted images. This inconsistency indicates that the perceived face-likeness of inverted images might not be entirely comparable to that of upright images. Future studies should consider alternative methods or additional instructions to ensure a more consistent interpretation of inverted stimuli, thereby enhancing the robustness of conclusions drawn from face-likeness ratings.

Last, future research should consider incorporating gaze tracking to understand the participants’ focus on specific facial regions during unconscious processing. This could provide a more comprehensive understanding of attention distribution and its impact on face detection.

## Conclusions

In conclusion, our findings indicate that binary face stimuli do not consistently trigger the inversion effect, suggesting limitations in unconscious facial perception. However, stimuli with higher face-likeness scores exhibited notably shorter BT, underscoring the influence of face-likeness on processing speed. This supports the hypothesis that certain visual information can rapidly bypass suppression and reach conscious awareness.

The LMM analysis further confirmed that as face-likeness increased, BT decreased, and as object-likeness increased, BT also increased, though the effect was weaker. This highlights the distinct influence of facial cues on accelerating perceptual awareness. Additionally, the lack of correlation between the number of black pixels in binary faces and BT clarifies that low-level features do not significantly affect these outcomes. Our results emphasize the role of top-down effects in early perceptual processing. As noted in previous studies, activation of the FFA and/or P1 in response to invisible faces reinforces the idea that face-like stimuli, even when imperceptible, can evoke neural representations in face-specific cortical areas. Overall, this study contributes to a deeper understanding of the mechanisms involved in facial perception, particularly under CFS, suggesting that the brain can extract and process facial information from minimal cues.

## Supplementary Material

Supplement 1
