# Supplementary material for: Facial ambiguity and perception: How face-likeness affects breaking time in continuous flash suppression
Source: J Vis. 2024 Sep 27;24(9):18. doi: 10.1167/jov.24.9.18 (PMC11437706; doi:10.1167/jov.24.9.18)
Supplement: Supplement 1 [file jovi-24-9-18_s001.pdf]

# Supplementary Information

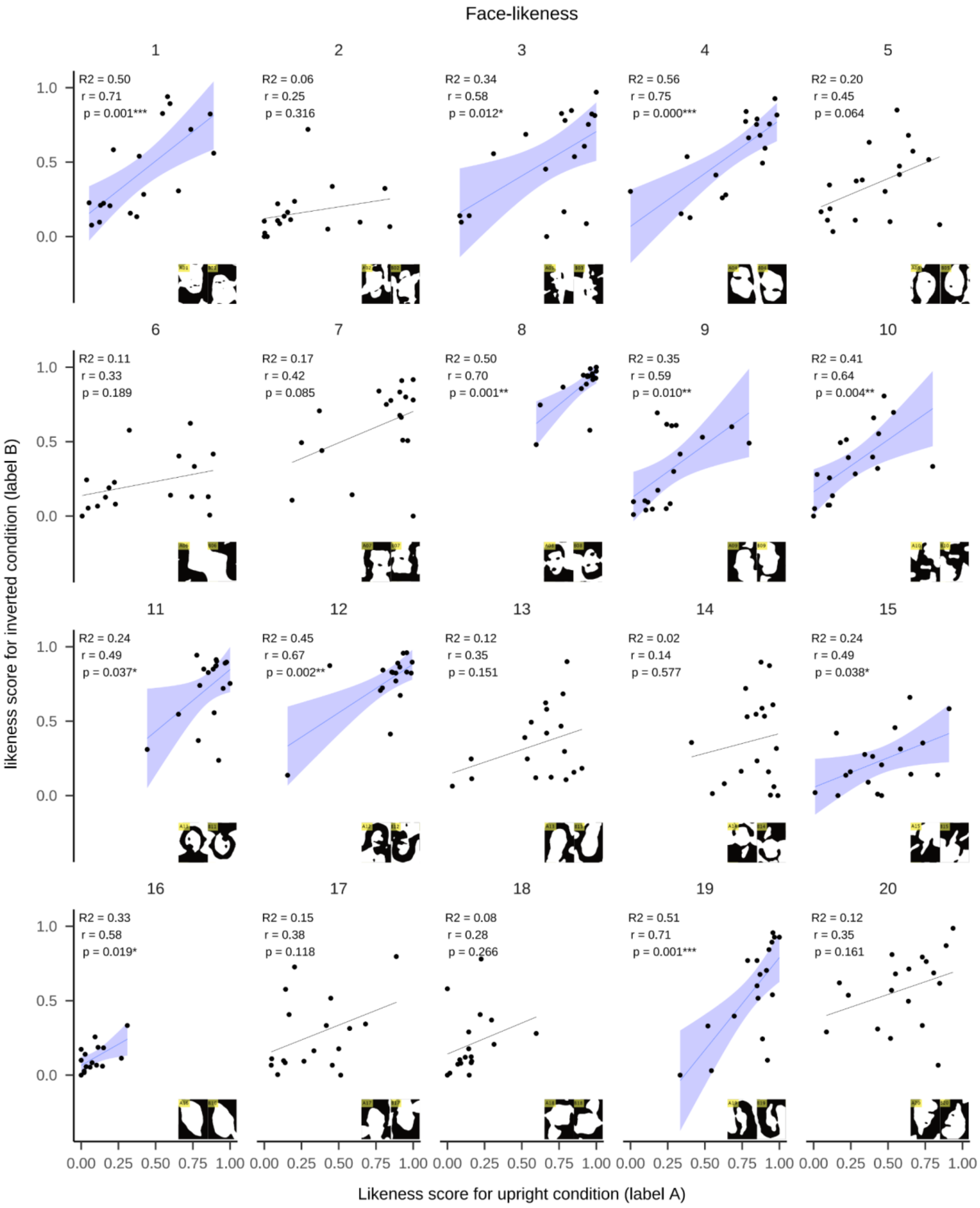


Figure S1 Likeness scores for upright (label A) and inverted (label B) conditions across different binary face stimuli. Each dot represents a participant, and each facet represents a different set of upright and inverted faces. Blue shaded areas represent 95% confidence intervals for sets with significant correlations ($p < 0.05$). The correlation coefficients ranged from 0.14 to 0.93 ($mean\ r = 0.57, mean\ R^2 = 0.36$).

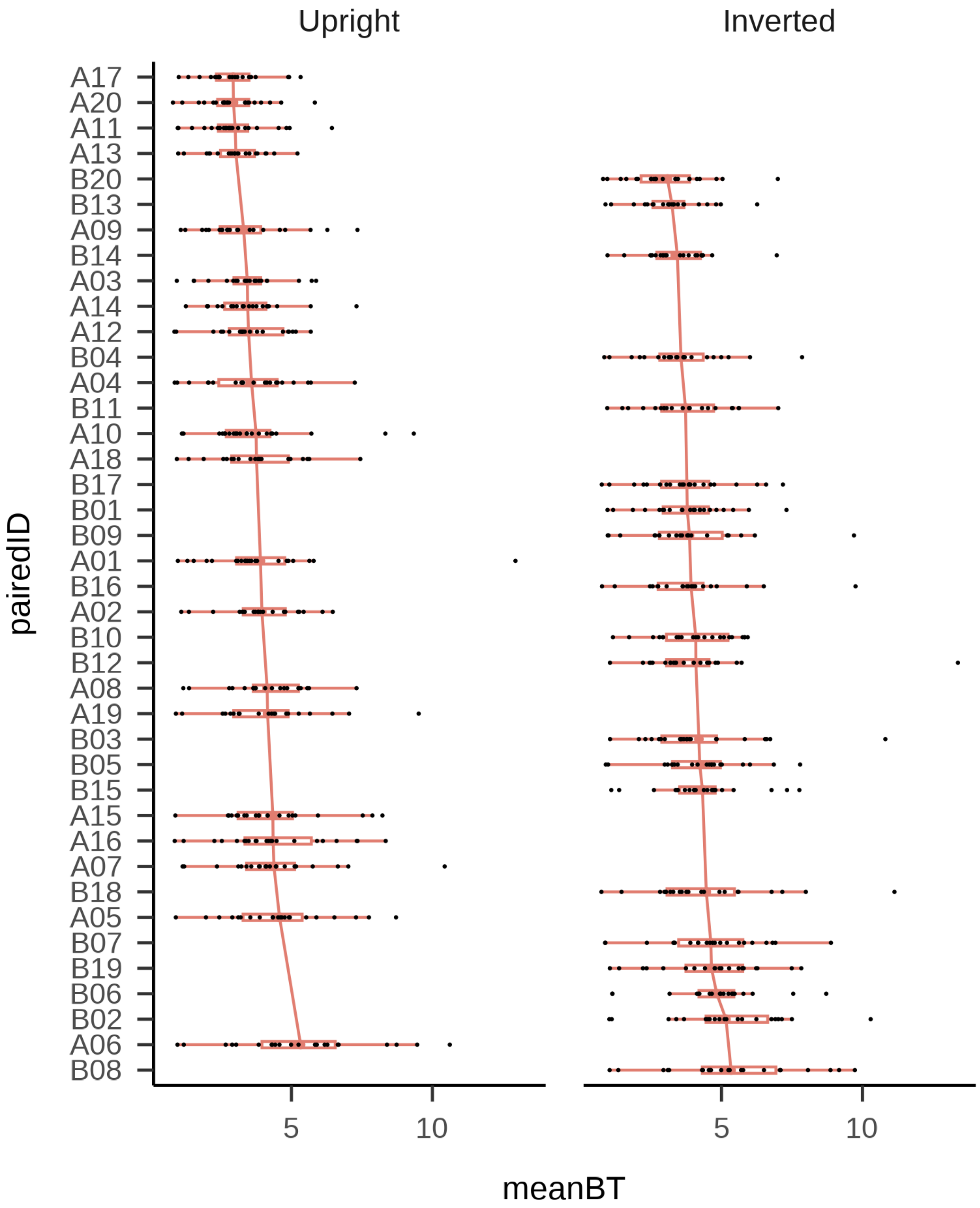


Figure S2 Analysis for grayscale stimuli data, comparing the mean BT (meanBT) for each paired ID across upright and inverted categories. Paired IDs were sorted with shorter mean BTs at the top among orientation categories.

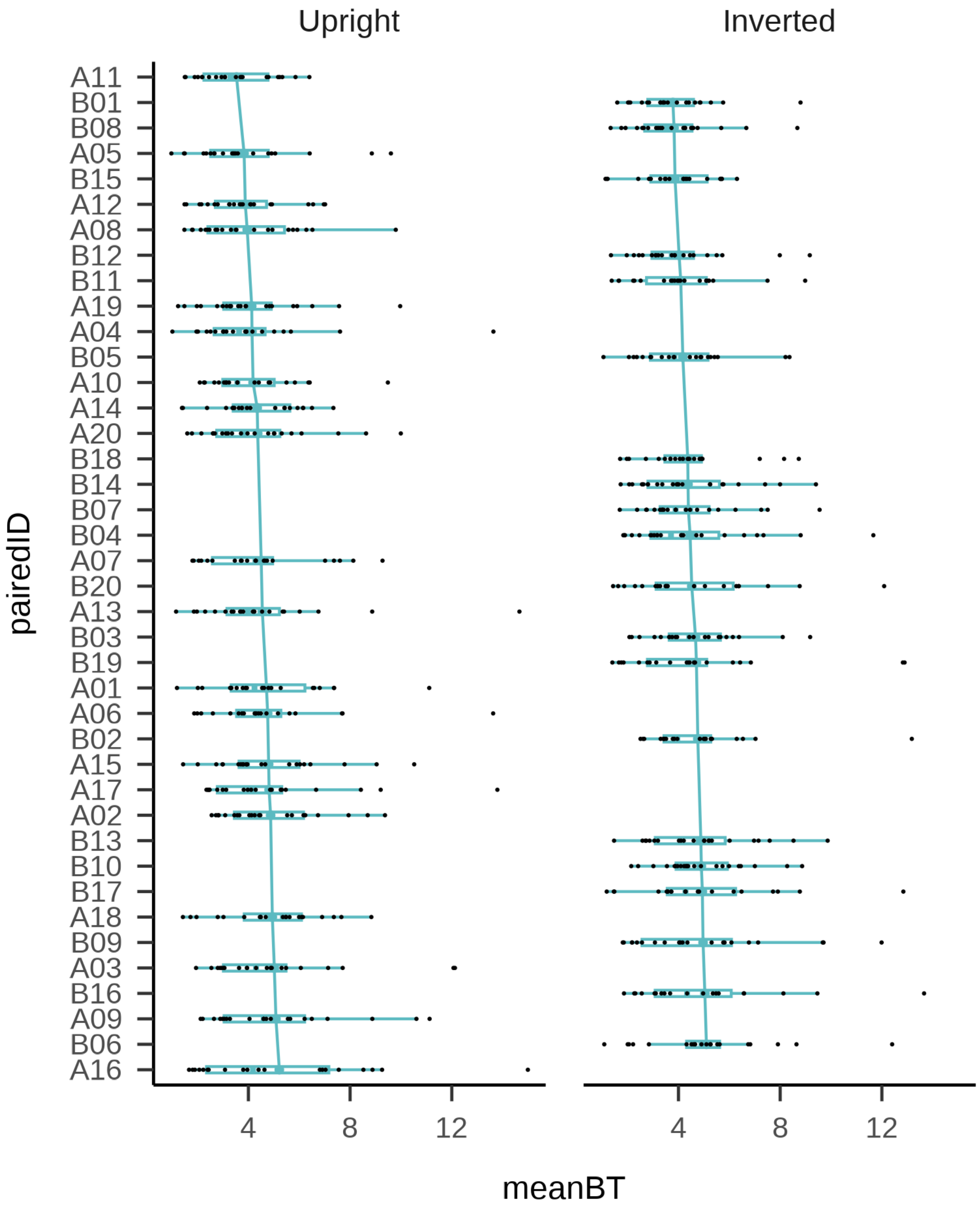


Figure S3 Analysis for binary stimuli data, comparing the mean BT (meanBT) for each paired ID (A is upright, B indicated inverted) across upright and inverted categories. Paired IDs were sorted with shorter mean BTs at the top among orientation categories.

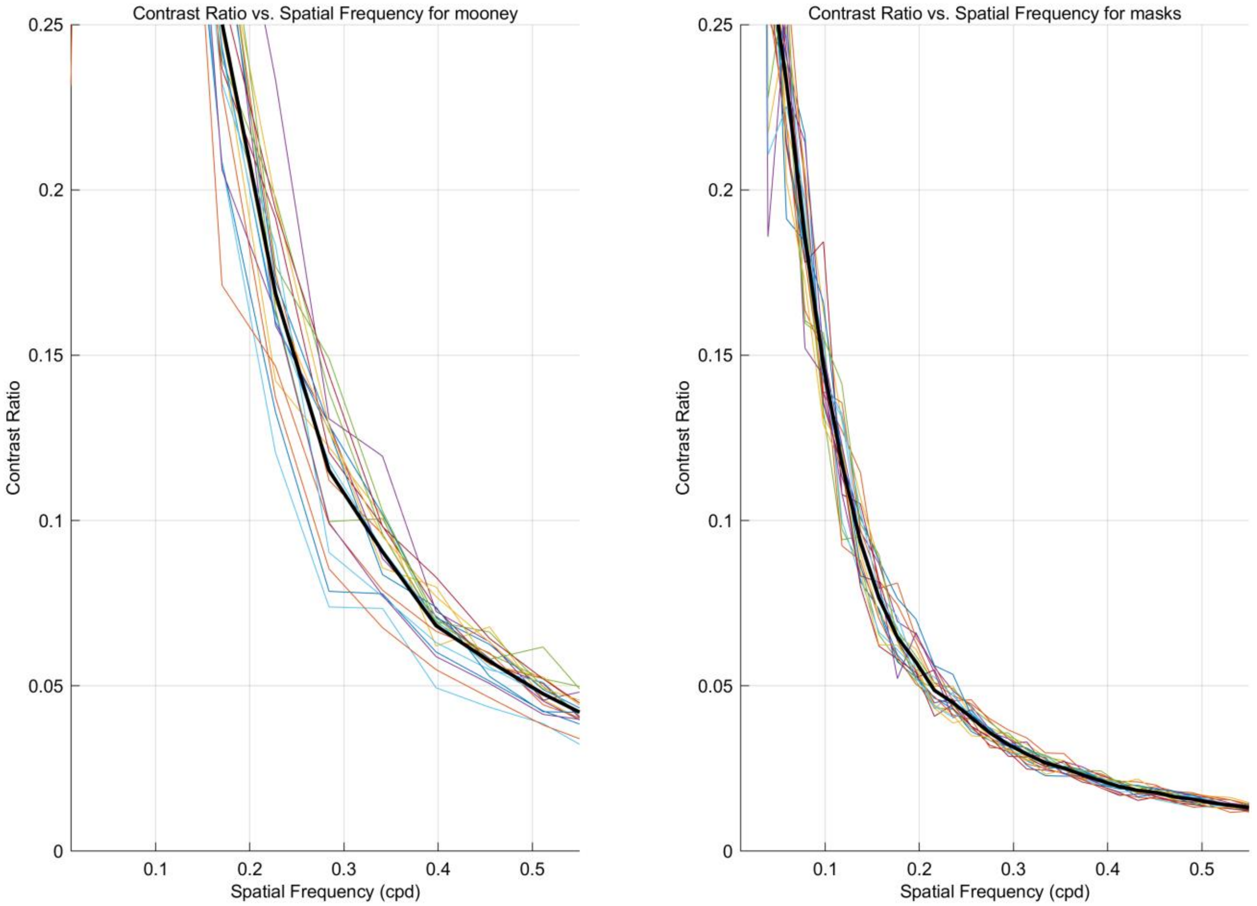


Figure S4 Contrast Ratio vs. Spatial Frequency for Mooney Images and Masks. The left plot shows the contrast ratio as a function of spatial frequency (in cycles per degree, cpd) for various Mooney images. The right plot displays the same analysis for different m

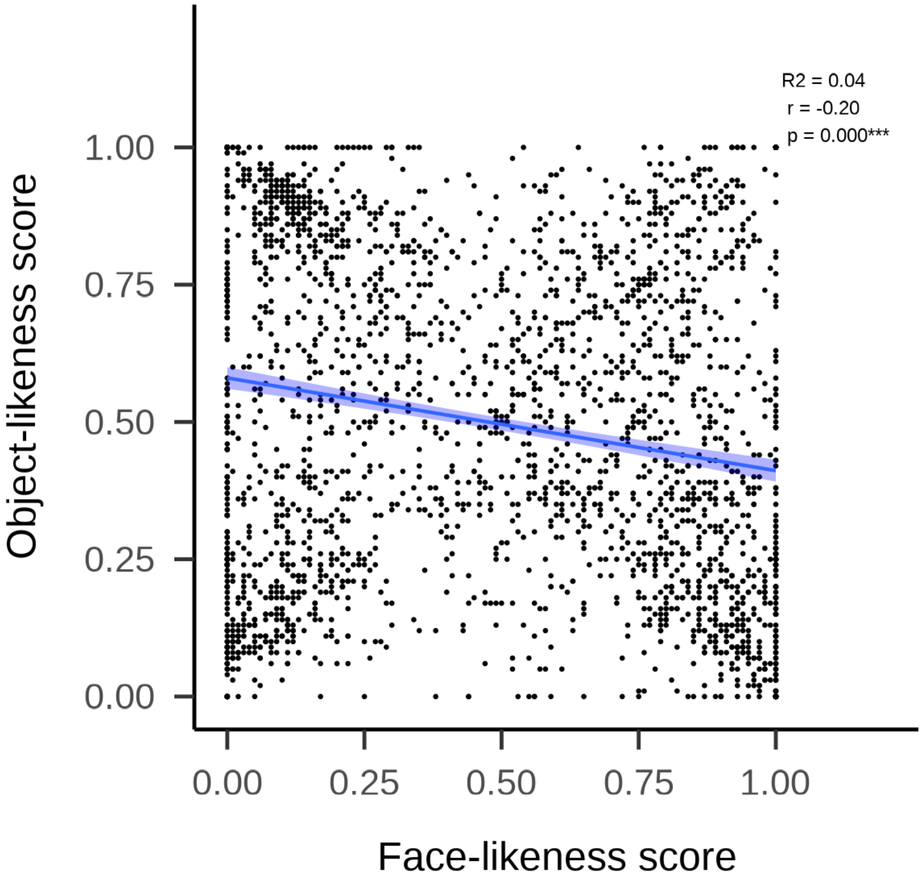


Figure S5 Likeness scores for face and object conditions across. Each dot represents a single response. Blue shaded areas represent 95% confidence intervals for sets with significant yet weak correlation $(r = -0.20, R^2 = 0.04, p < .000)$.
